# Quantifying the Epithelial-to-Mesenchymal Transition (EMT) from Bench to Bedside

**DOI:** 10.3390/cancers14051138

**Published:** 2022-02-23

**Authors:** Meredith S. Brown, Kristen E. Muller, Diwakar R. Pattabiraman

**Affiliations:** 1 Department of Molecular and Systems Biology, Geisel School of Medicine at Dartmouth, Hanover, NH 03755, USA; meredith.s.brown.gr@dartmouth.edu; 2 Department of Pathology, Dartmouth-Hitchcock Medical Center, Lebanon, NH 03756, USA; kristen.e.muller@hitchcock.org; 3 Norris Cotton Cancer Center, Dartmouth-Hitchcock Medical Center, Lebanon, NH 03756, USA

**Keywords:** epithelial-mesenchymal transition, EMT score, tumor heterogeneity, multiplexed immunofluorescence

## Abstract

**Simple Summary:**

Cell developmental programs used in wound healing and development such as the epithelial-to-mesenchymal transition (EMT) are frequently coopted by solid tumors to increase motility, plasticity, and invasive characteristics which promote metastasis. Identifying and quantifying the presence and extent of these programs can help to aid in patient prognosis and dictate therapeutic decision making. Here, we review the methods and findings to detect and quantify these cellular transitions in both laboratory and clinical settings.

**Abstract:**

The epithelial-to-mesenchymal transition (EMT) and its reversal, the mesenchymal-to-epithelial transition (MET) are critical components of the metastatic cascade in breast cancer and many other solid tumor types. Recent work has uncovered the presence of a variety of states encompassed within the EMT spectrum, each of which may play unique roles or work collectively to impact tumor progression. However, defining EMT status is not routinely carried out to determine patient prognosis or dictate therapeutic decision-making in the clinic. Identifying and quantifying the presence of various EMT states within a tumor is a critical first step to scoring patient tumors to aid in determining prognosis. Here, we review the major strides taken towards translating our understanding of EMT biology from bench to bedside. We review previously used approaches including basic immunofluorescence staining, flow cytometry, single-cell sequencing, and multiplexed tumor mapping. Future studies will benefit from the consideration of multiple methods and combinations of markers in designing a diagnostic tool for detecting and measuring EMT in patient tumors.

## 1. Introduction

During the progression of many solid tumors, cells at the primary tumor site undergo phenotypic changes in response to extracellular stimuli [1,2], one among these being an epithelial-to-mesenchymal transition (EMT). This embryonic developmental program increases invasive and migratory behavior that is advantageous to a metastasizing cancer cell [3], enabling them to disseminate to distant organs. Plasticity within this transition, including its reversal (mesenchymal-to-epithelial transition; MET) to regain epithelial and proliferative characteristics, has been demonstrated in metastatic colonization [4,5]. Indeed, patients whose tumors express high levels of EMT signatures have worse overall prognoses and increased rates of metastasis [6,7]. Previous works questioned the relevance of EMT in metastasis in breast [8] and pancreatic [9] cancers; however, these conclusions were based in either incomplete disruption of the intermediate EMT [10] or in lineage markers related to a highly mesenchymal state [11].

Critical to our understanding of epithelial–mesenchymal plasticity (EMP) and its underlying regulators is our ability to distinguish unique EMT states from one another for identification in vitro, in vivo, and in patient samples. EMP and heterogeneity have frequently been associated with poor patient outcomes [6,7]; however, no robust method for assessing either of these has been developed to complement histopathological assessment in the clinic setting. The presence and role of a variety of hybrid EMT states in disease progression and metastasis remains a lynchpin in EMT-based therapies. Current evidence suggests that rather than relying solely on an MET to revert from a fully mesenchymal state, metastasis may result from the high plasticity and adaptability of the intermediate or hybrid states [12,13], as observed by the presence of intermediate circulating tumor cell clusters (CTCs clusters) [14,15]. Regardless of a clear-cut mechanism, which is still currently in debate, the presence of an intermediate state appears to be critical to the formation of metastasis, either through plasticity or as a transitional state.

Here we review the various methods that have been utilized to identify the spectrum of E–M states within a sample, from flow cytometry to single-cell analysis of the intricate RNA and protein expression patterns found in mouse and human tumors. Ultimately, one or a combination of these methods could be applied to assess patient prognosis by providing rapid and comprehensive analysis of the EMT state and heterogeneity of tumors to inform disease aggression and treatment regimens.

## 2. Epithelial and Mesenchymal Markers

Several markers have been used over the years, firstly to determine the occurrence of EMT and, more recently, to distinguish various distinct states along the epithelial-to-mesenchymal spectrum. These markers are based on a range of properties, from those that inform stemness, to those that indicate morphological changes, and transcriptional regulators (EMT and MET TFs) of the transition. These markers have been used in various combinations, each with their own benefits and deficits, based on context, specificity, and ease of use (Table 1).

### 2.1. Morphological Markers of EMT

The first discovered and readily utilized markers for EMT relate directly to the morphological changes that cells undergo to enhance invasion and motility, such as loss of classical epithelial adherens tight junction proteins and gain of non-canonical alternative intermediate filaments. E-cadherin, a key component of adherens junctions, was first identified as lost in epithelial cells that gained invasive characteristics [16]. Other adherens and tight junctional components that are key indicators of the epithelial state include claudins, occludins, and catenins as well as desmosomal components, such as desmoglein and desmocollin [17]. On the other hand, markers, such as Vimentin [18,19], fibronectin, N-cadherin, and smooth muscle actin (SMA) [20], have all been used to identify mesenchymal-like cells as invasion and/or progression markers in multiple cancer types. Co-expression of one or more epithelial markers along with mesenchymal markers, e.g., E-cadherin and Vimentin, is frequently used to identify intermediate or hybrid EMT states [21]. Morphological markers serve as good tools for defining the EMT state because they reflect the morphology of the cells themselves. However, they are frequently expressed at varying degrees across the EMT spectrum and therefore make poor singular identifiers for any individual state.

### 2.2. Cell Surface Markers

While epithelial and mesenchymal markers, such as E-cadherin, vimentin, and fibronectin, serve to describe the internal cellular processes of EMT, they can be difficult to identify without permeabilization of the cell membrane, given their predominant intracellular localization. Consequently, cell surface markers and receptors have been adopted to identify and isolate E–M states while maintaining cell viability. EMT states were first stratified by CD44 and CD24 [22], and later by CD104 (ITGB4) [23], to identify tumorigenic populations of cells, linking the invasive and disease progressing nature of EMT to stem-like processes of cancer stem cells, particularly in breast cancer [24,25]. EpCAM, an epithelial cell adhesion molecule similar to E-cadherin, has been used in many ways to identify cells exhibiting an epithelial state, particularly CTCs [26], and as a marker for flow cytometry [27]. Notably, Pastushenko et al. [28] profiled a panel of cell surface markers to describe the transitions across an EMT, identifying a gating strategy using Epcam, CD106, CD51, and CD61 that accurately captured cells in an intermediate state. These will be discussed in more detail below. Cell surface markers are easy to access and utilize for cell sorting, but ultimately have proven inconsistent across tumor types and models for accurately defining EMT states.

### 2.3. Transcription Factors

In addition to morphological characteristics, gene regulators of EMT or MET, such as transcription factors, provide a finer metric for measuring the progression of a cell across epithelial and mesenchymal transitions, and have been reviewed extensively [29]. These markers provide specificity, particularly when paired with morphological features. Master EMT regulator TFs, such as Snail [30], Twist1/2 [31], and ZEB1 [32,33], were originally identified as repressors of E-cadherin and regulators of plasticity and EMT. PRRX1 has also been implicated in later EMT staged in colorectal [34], thyroid [35], and gastric [36] cancers. Conversely, OVOL1/2 are required for the suppression of EMT and induction of MET in breast [37] and skin [38] epithelial and cancer [39]. These markers, as well as others detailed in other reviews [29] have been used extensively in transcriptomics-based approaches to rank EMT [40] as well as image-based methods, described later in this review. Transcription factors provide detailed information on EMT state, especially when analyzed as a network. However, some TFs have tissue specific functions that can convolute a global EMT signature generated in this way.

**Table 1:** A summary of key EMT marker proteins and their use and relevance in determining EMT state.

Marker	Method(s)	Importance	Sample Type	Source(s)
E-cadherin	IF, transcriptional EMT scores	One of the first epithelial markers. Used for many analysis methods	Human tissue, human cell lines, mouse tissue, mouse cell lines	Behrens 1989 [16]
EpCAM	Flow cytometry, circulating tumor cells	Epithelial marker used in flow cytometry and CTC detection. Frequently lost early in EMT	Human tissue, human cell lines, mouse tissue, mouse cell lines	Riethdorf 2007 [26] Schnell 2013 [27]
Vimentin	Intracellular flow cytometry, IF, transcriptional EMT scores	Mesenchymal marker used in many analysis methods	Human tissue, human cell lines, mouse tissue, mouse cell lines	Sommers 1991 [18] Thompson 1992 [19]
CD44/CD24	Flow cytometry	Stemness markers first used to separate epithelial and mesenchymal states	Human tissue, human cell lines	Al-Hajj 2003 [22]
CD106/CD51/CD61	Flow cytometry	Used to segregate multiple EMT states	Mouse tissue, mouse cell lines	Pastushenko 2018 [28]
CD104 (ITGβ4)	Flow cytometry	An improved marker to replace CD24	Human tissue, human cell lines	Bierie 2017 [23]
Snail	IF, transcriptional methods, multiplexed image analysis	Transcriptional repressor of E-cadherin, responds to TGFβ signaling	Human tissue, human cell lines, mouse tissue, mouse cell lines	Cano 2000 [30] van Staalduinen 2018 [29]
Twist	IF, transcriptional methods	Hallmark EMT transcription factor	Human tissue, human cell lines, mouse tissue, mouse cell lines	Yang 2004 [31] van Staalduinen 2018 [29]
ZEB1	IF, transcriptional methods, multiplexed image analysis	Hallmark EMT-driving transcription factor, repressor of E-cadherin	Human tissue, human cell lines, mouse tissue, mouse cell lines	Guaita 2002 [32] Eger 2005 [33] van Staalduinen 2018 [29]
PRRX1	IF, transcriptional methods	EMT transcription factor prevalent in late EMT	Human tissue, human cell lines, mouse tissue, mouse cell lines	Takahashi 2013 [34] Hardin 2014 [35] Guo 2015 [36]
OVOL1/2	Transcriptional methods	MET transcription factor responsible for maintaining and epithelial state	Human tissue, human cell lines, mouse tissue, mouse cell lines	Roca 2013 [39] Watanabe 2014 [37] Li 2014 [38] van Staalduinen 2018 [29]

While several other cytoskeletal proteins, such as FSP1 [41] and α SMA [42], secreted proteins, including fibronectin [43] and MMPs [44], and epithelial junctional proteins, such as claudins and occludins [45], have been employed as EMT markers in different contexts, these have not specifically been used to identify intermediate/hybrid EMT states and could possibly highlight cells that reside in more extreme epithelial or mesenchymal states.

## 3. Model Systems Used to Study EMP

### 3.1. Cell Lines

Immortalized or cancer derived cell lines have been used for decades as models to understand cancer at a basic level. They are easy to work with, highly manipulable, and can provide a basis for testing novel drugs and therapies. Cell lines have been used to perfect many of the methods detailed below, including flow cytometry, immunofluorescence, and RNA-sequencing. Databases, such as the cancer cell line encyclopedia (CCLE) and ATCC, serve as repositories for data and frozen stocks of cell lines for research use. However, cell lines alone cannot recapitulate the complexities of an in vivo system, which can be achieved through orthotopic transplantation in mice, rats, and other model organisms. This model, therefore, serves as a necessary but simple steppingstone to understanding E–M heterogeneity and plasticity in patients.

### 3.2. Genetically Engineered Models

In efforts to recapitulate human tumor progression for laboratory study, many different non-human models have been generated that mimic aspects of patient disease to study the roles of EMT and MET in tumor development, progression, and metastasis. Most popular are genetically engineered mouse models (GEMMs), although zebrafish [46,47], drosophila [48,49], and sea urchin [50,51] models have been elegantly used to generate important insights in the field. These GEMMs provide an excellent framework for studying the metastatic cascade, allowing for spontaneous tumorigenesis in a specific tissue of choice, collection of organs and circulating tumor cells for basic research and diagnostic development, and testing novel drug targets in a complex living system. GEMMs have been used with great success to isolate and study EMP in vivo in breast [28], skin [28], pancreatic [52], and prostate [53] cancer. Additionally, immune compromised mouse models can host human-derived cell lines or patient-derived xenografts, which, when transplanted orthotopically into the tissue of origin, can recapitulate the nuanced disease of that individual for further study. Overall, mouse and other models of human cancer have been crucial in expanding on in vivo dynamics of the metastatic cascade where cell lines have fallen short.

### 3.3. Primary Human Tissue

Ultimately, the best tool for studying human disease is directly on human patient samples. This can be tumor or tissue biopsies either taken fresh or stored in a formalin fixative, as well as circulating tumor cells, cytological specimens, and secondary site biopsies, etc. However, acquiring patient samples and full patient data can be challenging and take years. Databases, such as The Cancer Genome Atlas (TCGA), contain complete sequenced genomes for thousands of primary patient samples and can be a helpful bioinformatic tool to begin transitioning from basic to translational research, such as validating cancer predictor genes or looking for large trends across many samples. EMP has been successfully identified, validated, and explored in patient cancers, including in circulating tumor cells [7,54,55], determining EMT gene signatures in primary tissue [56,57], and mapping EMT states at single-cell resolution [58,59], both substantiating its relevance in patient disease and opening new possibilities for diagnostic approaches.

### 3.4. Circulating Tumor Cells

Circulating tumor cells in the blood have served as a “window to cancer” for many years [60,61]. As a diagnostic tool, it is easy to implement on patients, requiring only a small blood sample, and can be used to screen tumor cells in a multitude of ways [62], including immunofluorescence [54], RNA in situ hybridization (ISH) [15], and RNA-sequencing [14]. CTC collection methods have also helped to validate the significance of an EMT in the metastatic cascade [15,54], as well as a possible requirement for the reversal, or MET, to colonize metastatic organs [63].

Traditionally, circulating tumor cells were harvested using the cell surface marker EpCAM [26], as most cancers of interest were epithelial in origin. However, EpCAM is lost during an EMT [64], leading to a misrepresentation of CTCs collected by this method. Indeed, even when captured with an EpCAM retrieval method, CTCs in breast and prostate cancer patients were found to co-express epithelial and mesenchymal markers in progressive disease [6], bringing up the question of how many EpCAM-negative mesenchymal cells were missed in the analysis. In response, other microfluidics-based methods of CTC capture have been adopted [65,66], although EpCAM-based methods still dominate patient diagnostics [67]. Non-specific capture methods have identified relatively equal populations of epithelial, intermediate, and mesenchymal CTCs, defined by EMT markers, such as E-cadherin and vimentin; however, multiple studies have found a correlation between high presence of mesenchymal CTCs and worsened patient prognosis [7,63]. Along with CTCs, microfluidics devices have also identified circulating tumor cell clusters (CTC clusters) which, although rare, are much more potent metastatic seeders than individual CTCs alone [14]. Under unbiased collection, identification and classification of CTCs and CTC clusters by microfluidics is a powerful diagnostic tool that can be combined with a multitude of other methods to understand EMP and its role in the metastatic cascade.

## 4. Methods

### Flow Cytometry

**Figure 1:**
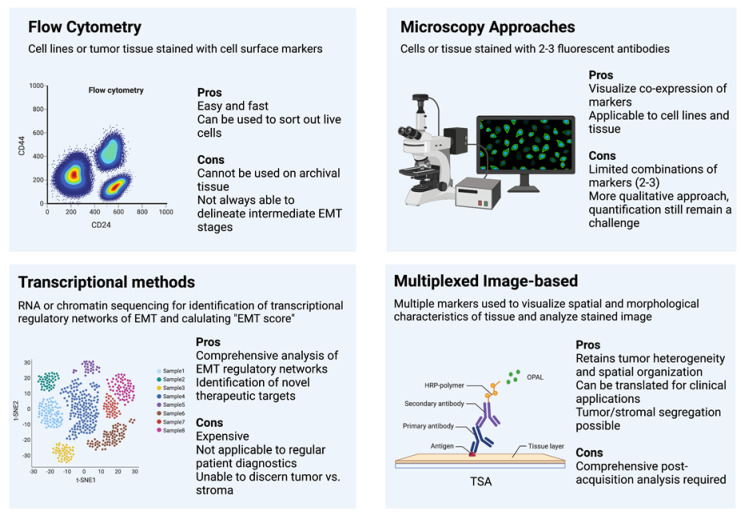
Methods of Assessing EMT.

Flow cytometry techniques can easily and readily detect cell populations expressing a combination of cell surface markers. Further, live cell populations can be sorted based on marker expression via fluorescence assisted cell sorting (FACS) for further study. This has made flow cytometry a very popular and easily applicable resource in many early studies defining E–M states. The invasive nature of cells undergoing an EMT elicited a natural link to cancer stem-cell like states, prompting these tumorigenic populations of cells to be initially isolated and described as CD44^hi^/CD24^lo^ [22], ALDH+ [68] stem-like populations, and later linked to the EMT process [24,25]. Flow cytometry has provided a means of differentiating epithelial (EpCAM+/CD24^hi^/CD44-) and mesenchymal (EpCAM-/CD44^hi^/CD24-) in multiple cancer cell lines [69,70] as well as the breast [71] and prostate [53] mouse model to delineate differences between these states, understand the unique mechanisms that control EMT and MET, and determine their various roles in disease progression and the metastatic cascade. Further endeavors to increase the flow sorting sensitivity of E–M states has led to the discovery of novel EMT cell surface markers, such as CD104 (ITGB4) [23] as a supplement in addition to CD44/CD24 to define intermediate EMT states with cancer stem cells properties within human tumors, as well as combinations of EpCAM, CD51, CD61, and CD106 [28] to isolate multiple transitional intermediate/hybrid EMT states from mouse tumor models (Figure 1).

These works and the application of flow cytometry established a link between the hybrid or intermediate state and increased stemness and decreased patient prognosis using several cell surface markers; however, this technique is unable to consider the expression of intracellular markers, such as vimentin or ZEB1, which require cell permeabilization. In addition, this technique can only be used in live tissue, making it more challenging to study patient samples, which are often archival formalin-fixed paraffin embedded (FFPE) tissue. Additionally, evidence from our recent study on EMT states suggests that canonical cell surface markers (CD44, CD104, and EpCAM) are not sufficient to separate distinct intermediate states from one another [59]. Indeed, Pastushenko et al. [28] relied upon the co-staining of four markers and precise gating strategies to adequately separate these states. Thus, flow cytometry presents an excellent approach for basic biological analysis of E–M plasticity but has few applications for direct use on archival patient tissues.

## 5. Immunohistochemistry and Fluorescence

Immunohistochemistry is the most common form of immunostaining and has been used for decades to detect and label antigens in tissue sections [72]. Hematoxylin and eosin (H&E) staining for DNA and proteins, respectively, is the principal method for histological assessment of tumor grade and histological subtype. In addition to assessing tumor grade and histologic subtype, IHC staining for other biomarkers is routinely performed by pathologists for certain tumors, such as hormone receptors, HER2, and Ki67 in breast cancer, to provide prognostic and predicative information, and to stratify tumors into molecular intrinsic subtypes [73,74].

Immunofluorescence (IF) staining for EMT markers, particularly E-cadherin and Vimentin, has frequently been used alongside other methods for visualizing the co-expression of epithelial and mesenchymal markers as well as discerning the sub-cellular localization of proteins. However, it is rarely used as a comprehensive method for defining EMT states, owing, in part, to the limitation of fluorescence wavelengths that only allow visualization of a limited number of markers simultaneously. Efforts to combine changes in cell morphology with E-cadherin/Vimentin IF staining in a predictive EMT model have been partially successful in cell lines [75]; however, the application of this predictive model in vivo remains unclear. An immunofluorescence microscopy assay for cytoskeletal remodeling elements [76] has been successfully implemented as a readout for EMT disruption to screen a panel of transcription factor-targeting siRNAs to determine transcriptional nodes that control EMT [77], which can be useful for easy drug treatment and screening for future testable therapeutics targeting EMT. Moreover, recent work has combined fluorescent lineage tracing with intravital live microscopy to visualize and trace early and late EMT states in the primary tumor and metastatic sites, providing a much needed look inside the dynamic processes of tumor progression and EMT [13]. Combined immunofluorescence with other techniques, such as cell morphology or single-cell segmentation, has distinct advantages over bulk flow or sequencing by maintaining tumor architecture and spatial organization in the tissue. However, the limiting number of probes in classical IF presents the same drawbacks as flow cytometry and may be insufficient to describe the complexity of E–M states (Figure 1).

## 6. Transcription-Based Methods

RNA-based, and later chromatin-based, methods of assessing the EMT state have two main goals: to generate an EMT gene signature, or to characterize EMT gene networks across a spectrum of samples. This can be done in a variety of ways, although the goal is generally to further basic knowledge rather than apply directly to patients.

Bulk RNA-seq has been repeatedly used to generate EMT gene signatures or “EMT scores” to help standardize and define entrance into an EMT [56] or partial EMT states in many cancers [57] and correlate that gene signature with poor patient prognosis. This is useful in understanding the connection between EMT gene signatures and patient prognosis as well as defining EMT states for new model systems [59]. However, many groups have put forth their own signature or method for ranking EMT [40,56,57,78], calling into question a standardized signature for ubiquitous use. In a more exploratory approach, RNA-seq has been used to interrogate EMP using either isolated clonal states within an EMT [28,59], or an EMT induction and withdrawal (MET) time course. These experiments served to delineate the specific gene networks that are active during the transition from epithelial to mesenchymal and back [77,79] and to help distinguish targetable transcriptional networks in aggressive or metastatic cell states. Combinations of EMT induction and siRNA knockdown of EMT target transcription factors identified control nodes, such as TEAD2, FOSL2, SP1, and others that had not been previously associated with EMT [77].

Other approaches to visualize RNA expression, such as fluorescence in situ hybridization (FISH) probing for a panel of EMT markers, have had success in assessing EMP at a single-cell level in circulating tumor cells [15] before the more widely accepted single-cell sequencing approaches were robust enough to be used in this context.

Single-cell RNA sequencing has been particularly vital in assessing EMT in tumors or cells where the bulk RNA signature may not be sufficient to describe the heterogeneous populations within each sample. This can be applied to CTCs [55], tumor cells [80], or as part of induced time course [79,81] to delineate EMP in these samples as well as interrogate EMP and heterogeneity at multiple points during metastasis, including the primary tumor, CTCs, and metastatic sites, to see how EMT states may work individually or cooperatively to promote metastasis.

A leap in the field came from non-specific sequencing of accessible chromatin (ATAC-seq), which revealed large scale chromatin modification in response to progression through an EMT [28,82,83], indicating that large transcriptional shifts may be controlled through a combination of epigenetic and transcriptional regulation. Recently, multiple efforts have taken a multi-omics approach, combining RNA-seq and ATAC-seq to determine these combined epigenetic and transcriptional regulatory proteins, such as CTCF, the AP-1 complex, and the RUNX transcription factor family [59,84,85].

While transcription-based approaches have provided a wealth of data and greatly contribute to the understanding of EMT, MET, and the metastatic cascade, the cost of sequencing, the processing times, and the inability to segregate tumor from stroma still make it inapplicable to assess patient samples for routine diagnosis. However, these comprehensive analyses have pinpointed specific EMT indicators for further and more directed approaches, such as multiplexed staining (Figure 1).

## 7. Multiplexed Image-Based Methods

Akin to immunohistochemistry, image-based methods of assessing tumors have distinct advantages for patient diagnosis. They are relatively easy, can be done with high throughput, and most importantly, retain spatial organization and heterogeneity of the original tumors. However, immunohistochemistry or even immunofluorescence struggles to describe the complexity of patient tumor states that may affect disease progression and metastasis, particularly epithelial to mesenchymal plasticity. Previous efforts have combined immunohisto-fluoresence for E-cadherin and Vimentin with high content screening (HCS), introducing a method that combines cell segmentation, morphological evaluation, and marker expression to determine nuanced EMT states within a tumor sample at a single-cell level [75]. This has been implemented with various other probes and image analysis software to combine immunofluorescence and morphological features into a reliable patient diagnostic tool [86,87]. However, this method is limited by the number of markers that can be used. Others, in efforts to combat this issue, have relied on mass cytometry time courses to map changes and co-expression of E-cadherin, Vimentin, CD44, CD24, and others in individual cells undergoing a TGF-β-induced EMT in lung cancer [58]. Similar methods have been used for multiplexed identification and stratification of heterogeneity in breast cancer patient samples with 35 different markers [88]. Even newer technologies, such as Nanostring DSP, present exciting new platforms for high-plex spatial imaging of RNA and/or proteins. This platform is becoming increasingly useful to deconvolute tumor heterogeneity and the tumor microenvironment (TME) of specific tumor types [89]. These approaches rely on precise image analysis software that has only recently become sensitive enough to reliably segment individual cells and deconvolute multiplexed staining approaches. Considering these technological advances, image-based approaches to quantify EMT progression in tumors are becoming more popular and easily implementable; these technologies have been reviewed extensively elsewhere [90]. Recently, we have employed a multiplexed, multi-round tyramide signal amplification (TSA) staining method using six canonical EMT markers that was used with cell segmentation and morphological features to define an EMT heterogeneity score and overall tumor EMT score in a model system of EMT, and further validated in a cohort of breast cancer patient samples [59]. Notably, this method was the first to reliably segment out stromal tissue, such as fibroblasts, which can surround tumors and frequently express Vimentin and ZEB1, mesenchymal markers that would skew an analysis of tumor composition. When implemented with widely practiced immunohistochemical approaches to determining tumor grade and composition, this method can help to elucidate the complexity of patient tumor heterogeneity and EMT state in the clinic to better inform prognosis and treatment regimes (Figure 1).

## 8. Conclusions

Endeavors to characterize, quantify, and stratify epithelial–mesenchymal cell states in research models and patient samples has spanned decades. With each technological leap, the field gains more knowledge and insight into the markers and methods that can best and most simply stratify these phenotypic cell states. While no method is obsolete, some, such as immunohistochemistry, have made way for more complex and descriptive methods, such as multiplexed immunofluorescence. Ultimately, when patient diagnosis is the goal, approaches should be tailored for these specific needs, such as ease of use, number of samples at a time, cost, and accurate resolution of tumor tissue and individual cells. Flow cytometry and immunohistochemistry are simple and easy to implement, but lack the complexity and standardization required to reliably identify and score EMT states in patient samples from many different tumors. Transcriptional and chromatin-based methods provide this complexity and have pulled back the veil on the intricate transcriptional and chromatin regulatory networks that controlled epithelial–mesenchymal plasticity. However, they are difficult to implement on fresh samples and are quite cost prohibitive. For this reason, they remain a strong tool for use on test cohorts and in vitro or in vivo models of EMT but are unlikely to be adopted for routine diagnostics. Circulating tumor cells have been excellent diagnostic tools in patients for many years as they are easy to sample and provide a heterogeneous window into the tumor itself. CTCs also provide a background for testing many prognostic tools and have been tested out with many methods, past, present, and future. Image-based approaches have built upon what the field has learned about most descriptive and succinct E–M markers, as well as tissue and single-cell segmentation to create a robust tool to apply across many patient samples and in many contexts. In the future, such a method can be used to complement histopathological assessment in a clinical setting to provide a rapid and comprehensive analysis of E–M heterogeneity and the EMT tumor score to predict disease progression and inform treatment regimens.
